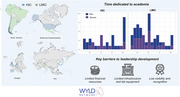# Understanding the challenges faced by dementia researchers: Findings from a global survey on capacity building

**DOI:** 10.1002/alz70860_104349

**Published:** 2025-12-23

**Authors:** Florentina Morello Garcia, Nicolas Corvalan, Micaela Maria Arruabarrena, Maria Florencia Clarens, Greta Keller, Loana De Los Santos, Maria Eugenia Martin, Ricardo Allegri, Cristiano Aguzzoli, Livia Amaral, Carolina Agata Ardohain Cristalli II, Bruna Bellaver, Merci N Best, Madeleine Bloomquist, Igor C. Fontana, Kevin Chen, Neha Dubey, Cynthia Felix, Diego Fernandez Slezak, Indira Ruth Garcia Cordero, Micaela A Hernández, Ozama Ismail, Florence U Johnson, Arshia A Khan, Suelyn Koerich, Maria Celeste López Moreno, Pamela C.L. Ferreira, Nahuel Magrath Guimet, Marina Scop Madeiros, Markley Silva Oliveira, Guilherme Povala, Andreia Rocha, Matheus Scarpatto Rodrigues, Emma Ruppert, Kaitlin Seibert, Carolina Soares, Ezequiel Ignacio Surace, Hannah M Wilks, Tharick A Pascoal, Jorge J. Llibre‐Guerra, Luc¡a Crivelli

**Affiliations:** ^1^ Institute of Neurosciences (INEU), Fleni‐CONICET, Buenos Aires, Buenos Aires, Argentina; ^2^ Fleni, Buenos Aires, Buenos Aires, Argentina; ^3^ Fleni, Buenos Aires, Argentina; ^4^ Brain Institute of Rio Grande do Sul (InsCer), Porto Alegre, Rio Grande do Sul, Brazil; ^5^ University of Pittsburgh, Pittsburgh, PA, USA; ^6^ University of Michigan, Ann Arbor, MI, USA; ^7^ Alzheimer's Association, Chicago, IL, USA; ^8^ University of Calcutta, Kolkata, West Bengal, India; ^9^ University of Buenos Aires, Ciudad Autonoma de Buenos Aires, CABA, Argentina; ^10^ Tanz Centre for Research in Neurodegenerative Diseases, University of Toronto, Toronto, ON, Canada; ^11^ University of Minnesota Duluth, Duluth, MN, USA; ^12^ University of Texas Health Science Center at Houston, Houston, TX, USA; ^13^ Institute of Basic and Applied Psychology and Technology (IPSIBAT), National University of Mar del Plata (UNMDP), Psychology Faculty, Mar del Plata, Buenos Aires, Argentina; ^14^ University of Chicago, Chicago, IL, USA; ^15^ Washington University in St. Louis, St. Louis, MO, USA; ^16^ Washington University in St. Louis School of Medicine, St. Louis, MO, USA

## Abstract

**Background:**

Different global initiatives aim to support researchers in their fields of study. World Young Leaders in Dementia (WYLD) is an international organization dedicated to connecting and supporting young professionals in the dementia field. One of its missions is to promote the development of leadership skills through capacity building. This concept encompasses five key areas: education and training, funding and investment, infrastructure development, collaborations and networking, and community engagement. Understanding researchers' working and academic conditions is the first step toward implementing targeted interventions to foster their growth. The aim of this work is to describe the main challenges faced by dementia researchers globally.

**Method:**

A comprehensive survey was designed to gather insights from dementia researchers, exploring demographic information and five key domains of capacity building. It was disseminated through WYLD's membership list and partnerships with dementia‐focused organizations (e.g., ISTAART Professional Interest Areas, International Neuropsychological Society Special Interest Groups). A preliminary analysis was performed, focusing on education and training as well as funding and investment.

**Result:**

The participants (*n* = 125) were primarily women (69%) residing in low‐ and middle‐income countries (75.2%). Among respondents, only 39% hold full‐time academic positions, and just 20% dedicate 100% of their professional time to academia. A 65% report that the scientific system in their country is either underdeveloped or not prioritized as public policy. Furthermore, 93.7% state that funding sources—including salaries, grants, travel scholarships, and other forms of support—are scarce or nonexistent for early‐career researchers. However, 62.4% reported receiving specialized training in dementia, and 65.6% stated their groups provide regular learning opportunities. Finally, the primary barriers to leadership development were: limited financial resources, limited infrastructure, and low visibility and recognition. Figure 1 summarizes the main results.

**Conclusion:**

The findings underscore significant challenges faced by dementia researchers. Limited financial resources, inadequate infrastructure, and low visibility and recognition remain critical barriers to leadership development and professional growth. Nevertheless, many researchers reported access to specialized training and regular learning opportunities within their groups, providing a foundation for strengthening capacity‐building initiatives. Closing these gaps through targeted interventions is crucial to fostering the next generation of leaders in dementia research.